# Knowledge, Attitude and Behaviours towards Recommended Vaccinations among Healthcare Workers

**DOI:** 10.3390/healthcare5010013

**Published:** 2017-03-07

**Authors:** Giuseppe La Torre, Stefania Scalingi, Veronica Garruto, Marco Siclari, Massimiliano Chiarini, Alice Mannocci

**Affiliations:** Department of Public Health and Infectious Diseases, Sapienza University of Rome, 00185 Roma, Italy; stefaniascalingi@gmail.com (S.S.); veronicagarruto11@gmail.com (V.G.); info@marcosiclari.it (M.S.); massimiliano.chiarini@uniroma1.it (M.C.); alice.mannocci@uniroma1.it (A.M)

**Keywords:** knowledge, attitude, behaviours, vaccinations, healthcare workers

## Abstract

Healthcare workers (HCWs) are an important group of professionals exposed to biological risk during their work activities. So, the aim of this study is to perform a survey on the knowledge, attitude and behaviour of Italian HCWs towards the vaccinations recommended by the Ministry of Health. A cross-sectional study was carried out during the period September 2014–August 2015 in the Lazio region. The study was conducted by recruiting HCWs and biomedical students. The sample was comprised of 571 responders, of whom 12.4% were physicians, 18.9% were nurses, 34.3% were other HCW, and 34.3% were biomedical students (medical and nurses students). Hepatitis B virus (HBV) is perceived as a risk for personal health by 457 (80%) participants; TB is also worrying (434; 76%). Moreover, HBV (70.9%) and tuberculosis (TB) (79.2%) are perceived as a risk for health, while influenza is not considered so by most participants (46.2%). There is an underestimation of the role of influenza, perceived as a risk for 137 respondents (24%). The vaccination rate among these HCWs is highest for Hepatitis B virus (HBV) (82%), and lowest for influenza (28.5%) and varicella (40.3%). The vast majority of responders are in favour of HBV (77.8%) and TB (64.8%) vaccines. For other vaccinations there is less interest (between 33% and 40% for measles, mumps, rubella, pertussis and influenza). This study shows that knowledge of recommended occupational vaccinations is insufficient in HCWs, with few exceptions represented by HBV and TB. There is a need for novel approaches in this field, with the aim of enhancing vaccine coverage among HCW.

## 1. Introduction

Healthcare workers (HCWs) are an important group of professionals exposed to biological risk during their work activities due to contact with patients and materials that are potentially infected. In this field information, training and prevention are crucial. Vaccinations are the most important strategy for primary prevention.

In some cases, when a disease can be transmitted through humans, such as influenza or hepatitis B, the vaccine protects not only the HCW but also colleagues and patients to whom s/he could potentially transmit the infection [1,2]. However, the vaccination rates among healthcare professionals are not satisfactory, even if there is some evidence of efficacy [3,4,5].

In Europe, there are a lot of differences between the recommended vaccinations for HCWs, as well as differences in the implementation frame (mandatory or recommendation), target HCW groups and health-care settings [4,6,7,8].

In Italy the Ministry of Health recommends the following vaccinations for HCWs:

(1) Flu vaccination;
(2) Tubercular vaccination (BCG);
(3) Measles, mumps and rubella vaccination (MMR);
(4) Varicella vaccination;
(5) Pertussis vaccination;
(6) Hepatitis B vaccination.

So, the aim of this study is to perform a survey on the knowledge, attitude and behaviours of Italian HCWs towards the vaccinations recommended by the Ministry of Health.

## 2. Materials and Methods

A cross-sectional study was carried out during the period September 2014–August 2015 in the Lazio region. The study was conducted by recruiting 612 HCWs and biomedical students in the following settings: Teaching Hospital Umberto I Rome; Undergraduate course of Nursing at Pomezia, Sapienza University of Rome; S. Anna Hospital, Pomezia; Dono Svizzero Hospital, Formia.

The questionnaire, administered anonymously, contains biographical details (type of profession, age, gender, years of occupation) and a part concerning questions about knowledge, attitude and behaviours towards vaccine-preventable diseases. The following items (possible answers: yes/no) have been considered:

- Q1: “Which infectious diseases could be a risk for my health, if I am not vaccinated?”
- Q2: “Is exposure to the disease a concrete risk factor for the patient’s health?”
- Q3: “Does the disease X vaccine pose a risk to my health?”
- Q4: “Are the benefits of the disease X vaccine higher than the risks for me?”
- Q5: “Will the disease X vaccine protect my patients?”
- Q6: “For which disease have I been vaccinated?”
- Q7: “Have you had side effects from this vaccination?”
- Q8: “For which diseases is the National Health System (NHS) providing sufficient communication/information?”
- Q9: “For which diseases do you believe a vaccination should be mandatory for health care workers?”
- Q10: “For which diseases do you believe a vaccination should be mandatory for biomedical students?”
- Q11: “For which diseases do you believe a vaccination should be highly recommended, like for health care workers and students?”

The statistical analysis was conducted using a descriptive approach and a multivariate approach. For the first one the prevalence of an answer was reported for each item. Moreover, a multiple logistic regression analysis, considering all of the respondents who answered yes vs. all who answered no to the questions, was carried out for each item for each disease, taking into consideration the following variables as explanatory: type of profession (nurses, other professions, students vs. physicians as a reference group), gender (males as reference), age and years of occupation (both as quantitative variables) and type of structure (public vs. private, the latter as reference). Multicollinearity between type of profession and age was checked. The results of the multivariate analysis are presented for each variable as an Odds Ratio (OR) and 95% Confidence Interval (95% CI).

The statistical significance was set at *p* < 0.05.

The statistical analysis was carried out with SPSS, release 23.0. (IBM, North Castle, NY, USA)

All subjects gave their written informed consent for inclusion before they participated in the study. The study was conducted in accordance with the Declaration of Helsinki, and the protocol was approved by the Ethics Committee of Sapienza University of Rome (Prot. 16, 24 June 2014).

## 3. Results

The sample was comprised of 571 responders (response rate 93.3%), of whom 12.4% were physicians, 18.9% were nurses, 34.3% were other HCW, and 34.3% were biomedical students. Our respondents are mainly employed or are students in public bodies (76.7%). The mean (SD) and the median age of participants were 33.6 (SD 12.8) and 29 years, while the mean and median working age of participants were 13.0 (SD 11.4) and 10 years. Most of the responders were females (366, 64.1%).

### 3.1. Knowledge and Attitude

Hepatitis B is perceived as a risk to personal health by 457 participants (80%), as well as TB by 434 (76%). There is an underestimation of the role of influenza, perceived as a risk for 137 respondents (24%) (Table 1).

Concerning the question “Is exposure to the disease a concrete risk factor for the patient’s health?”, again hepatitis B (70.9%) and TB (79.2%) are perceived as a risk to health, while influenza is not considered so by most participants (46.2%) (Table 1).

As concerns the question “Does the disease X vaccine pose a risk to my health?”, there is a general agreement that vaccines are safe. However, vaccines against TB and flu are considered a risk to health by 14.9% and 14.7% of respondents, respectively, while the vaccine against pertussis is perceived as the safest (considered a risk by only 7.4%) (Table 1).

Vaccinations are seen as a positive preventive tool by more than 50% of participants (Table 1). For 73.2% the benefits of vaccination against HBV are higher than the risks for HCWs.

Concerning the question “Will the disease X vaccine protect my patients?”, HBV vaccine is believed to be the most efficacious for protecting patients’ health (70.1%), while vaccination against flu is considered to be the worst (positive answers for 54.1% of the sample) (Table 1).

Concerning the mandatory and recommended use of vaccines for HCWs, in relation with issue 8 “For which diseases do you believe a vaccination should be mandatory for health care workers?” the vast majority of responders are in favour of HBV (77.8%) and TB (64.8%) vaccines. For other vaccinations there is less interest (between 33.8% and 40.3% for measles, mumps, rubella, pertussis and influenza) (Table 1). More or less the same results are reported for the mandatory vaccinations for biomedical students. Again, the vast majority of responders are in favour of HBV (75.7%) and TB (62.9%) vaccines. For other vaccinations there is less interest (between 33.5% and 38.9.3% for measles, mumps, rubella, pertussis and influenza) (Table 1).

Finally, for the question “For which diseases do you believe a vaccination should be highly recommended, like for health care workers and students?”, HBV vaccination was highly recommended for 71.5%, while they are more sceptical with regards to varicella vaccination for which the prevalence was the lowest (34.9%) (Table 1).

### 3.2. Behaviour

As concerns issue 6—“For which disease have I been vaccinated?” the situation is completely different for the different types of diseases. The vaccination rate is highest for HBV (82%), and lowest for influenza (28.5%) and varicella (40.3%) (Table 1).

In relation to safety issues after the vaccine, three (0.5%) HCWs reported side effects after measles vaccine, two (0.4%) after rubella and pertussis vaccine, 4 (0.7%) after TB vaccine, five (0.9%) after mumps, varicella and flu vaccines, and eight (1.4%) after HBV vaccine.

Concerning the communication/information role of the NHS, the HCWs express a variety of opinions, with the exception being influenza (37.1%). According to the responders, the NHS is not performing a good communication concerning pertussis (11.2% of positive answers), followed by varicella (11.7%), mumps (13.5%), rubella (14.9%) and measles (17.7%) (Table 1).

### 3.3. Multivariate Analysis

As concerns hepatitis B (Table 2), the variable age is significantly associated with seven out of 11 items considered, with increasing age as a protective factor (meaning that younger healthcare workers were more likely to believe that Hepatitis B could be a risk to their health), while on the other hand years of work is a risk factor for five items (meaning that HCWs with more years of work were more likely to believe that Hepatitis B could be a risk to their health). Generally speaking, being a nurse or other professionals, as opposed to a physician, is a protective factor for the item under consideration (seven out of 11), and students have the same pattern only for a single item (Will the disease X vaccine protect my patients?). Females represent a risk factor for four items, while being in public employment is a risk factor for a single item (For which diseases is the National health system providing sufficient communication/information?) and a protective factor for two items.

In relation to TB (Table 3), the variables age, years of work, nurses and working in public bodies are not associated with any items. Other professions, compared to physicians, are protective factors for the item “Is exposure to the disease a concrete risk factor for the patient’s health?” while being students is a risk factor for a single item (For which diseases do you believe a vaccination should be mandatory for health care workers?). Females represent a risk factor for three items.

As concerns measles (Table 4), the variable age is significantly associated with six out of 11 items considered, with increasing age as a protective factor for five; on the other hand, years of work is a risk factor for four items and protective for one item. Generally speaking, being a nurse or other professionals, as opposed to a physician, is a protective factor for the items under consideration (eight out of 11), and students have the same pattern only for three items. Females represent a risk factor for a single item (For which diseases do you believe a vaccination should be mandatory for health care workers?), while being in public employment is a risk factor for four items.

Considering influenza (Table 5), the variable nurses and other professions, compared to physicians, is a protective factor for the items under consideration (five and nine out of 11, respectively), and students have the same pattern only for one item (For which diseases do you believe a vaccination should be mandatory for biomedical students?). Being employed in a public body is a risk factor for two items, and protective for a single item (Does the disease X vaccine pose a risk to my health?).

As far as concerns varicella (Table 6), the variables age and years of work are significant factors for one or two issues, respectively. Nurses, other professions and students, compared to physicians, are protective factors for one, three and two items under consideration, respectively, and all these categories are risk factors for the item “For which disease have I been vaccinated?” Being employed in a public body is a risk factor for a single item (Which infectious diseases could be a risk for my health, if I am not vaccinated?).

Concerning pertussis (Table 7), the variables age and years of work are significant factors for five and four issues, respectively. Nurses, other professions and students, compared to physicians, are protective factors for seven, six and one item(s) under consideration, respectively. Being in public employment is a risk factor for two items (For which disease have I been vaccinated? and For which diseases do you believe a vaccination should be mandatory for health care workers?).

In relation to pertussis (Table 8), the variables age and years of work are significant factors for eight and four issues, respectively. Nurses, other professions and students, compared to physicians, are protective factors for six, six and one item(s) under consideration, respectively. Being employed in a public body is a risk factor for two items (For which disease have I been vaccinated? and For which diseases do you believe a vaccination should be mandatory for health care workers?).

Finally, concerning rubella (Table 9), the variables age and years of work are significant factors for six and two issues, respectively. Nurses, other professions and students, compared to physicians, are protective factors for six, five and one item(s) under consideration, respectively. Being in public employment is a risk factor for three items (For which disease have I been vaccinated? and the two items concerning the mandatory vaccinations for HCWs and biomedical students).

## 4. Discussion

This study reveals that knowledge of recommended occupational vaccinations is insufficient in HCWs, with a few exceptions represented by HBV and TB. These results are in agreement with a couple of previous studies [9,10].

Our HCWs are aware that they are particularly exposed to vaccine-preventable diseases and can play a role in hospital transmission, which makes them an important target group for vaccination [11]. This fact needs to be addressed particularly for all HCWs, with the exception of medical doctors.

The scientific literature reports many examples of the relationship between HCWs and vaccination knowledge and needs. As concerns influenza, in a recent Italian study Albano et al. demonstrated that healthcare workers are not fully aware of the mode of transmission; HCWs with good knowledge of this issue were those who mostly read pertinent scientific literature [12].

The Italian Ministry of Health recommends flu vaccination with the aim of putting a barrier to the spread of the infection between patients and HCW and vice versa; this is particularly true if the patients are fragile [13]. However, flu vaccination among Italian HCWs is confirmed to be far below the satisfactory levels seen in the United Kingdom, Germany, France, Canada and Australia (prevalence of coverage between 28% and 51%), with a pooled prevalence of vaccinated of 11% and 15% among nurses and physicians, respectively [1,14]. Even if in some countries flu vaccination among HCW is low [15,16,17,18], there is evidence that flu vaccination among HCW decreases the risk of influenza-like illnesses in inpatients admitted to hospital for acute diseases [19], and reduces absenteeism due to respiratory infections [20].

HCWs in this survey gave particular attention to TB, even if in Italy the incidence rate of this disease is fewer than 10 cases per 100,000 inhabitants, and this country is classified by WHO as a low-incidence country. TB vaccination is mandatory for HCWs at a high risk of getting the disease [21]. Little attention is given by HCWs to vaccination against measles, mumps and rubella, as well as against varicella, while these are fundamental for susceptible HCW since in recent years the epidemiology of these diseases has changed, involving more and more adults and older people instead of children [22,23,24,25]. The vaccination rate against pertussis is also low among HCWs, as a recent review demonstrated. This vaccination is particularly important for HCWs who work in close contact with infants, children and pregnant women [26].

As concerns HBV vaccination, we can say that it needs to be implemented in countries where the incidence, as well as the probability of being infected if exposed, is high [27]. In Italy this vaccination has been mandatory for newborns since 1991, while it is recommended for HCWs. Among biomedical students, there is evidence that an association between the number of doses and levels of HBS-ab does exist [28].

Concerning the ethical aspects, some authors are in agreement with the fact that there is a moral imperative, not only for the general population [29], but also for HCWs to be immune and for healthcare bodies to require HCWs to be vaccinated, especially workers who are in contact with patients at high risk of infection [30]. This opens a window onto two related issues: the workplace and the mandatory implementation of vaccinations among HCWs. Concerning the first issue, one must recognize that HCWs are at increased risk for exposure to vaccine-preventable diseases (VPDs) in the workplace, so the reason for immunizing HCWs is based on the need to protect them and, indirectly, their patients from healthcare-associated VPDs [31].

There is consistent evidence showing that gaps in knowledge and false perceptions about VPDs and vaccines are the most common barriers to vaccine uptake in HCWs. Moreover, we need to consider that many countries have immunization recommendations in place for HCWs. On the other hand, no universal policies do exist and a lack of homogeneity between countries is present concerning the type of vaccines, schedules, frame of implementation (recommendation or mandatory), and target categories of HCWs [31].

Systematic reviews show that higher awareness and evidence-based beliefs of HCWs toward vaccination are associated with greater intentions to vaccinate [32], and in that case the role of vaccine education is crucial to avoid communication barriers [33].

In relation to the mandatory vaccination for HCWs, according to Maltezou and Poland, it should be considered that all infectious diseases can be transmitted to susceptible patients, such as hepatitis B, influenza, measles, mumps, pertussis, rubella and varicella [31]. However, the probability of adherence to mandatory vaccinations by HCWs is still uncertain and seems to be VPD-specific [4]. In practice, mandatory policies approved at the national level in Europe were only rarely implemented. More pragmatically, while guidelines for HCW vaccination, but not mandatory policies, have been adopted all over Europe, there is evidence that recommendations work better if they are focused on specific HCW groups and appropriate diseases (i.e., hepatitis B, influenza and measles) [34].

The present study has strengths and limitations. As strengths go, this represents a large survey involving almost 600 HCWs, interviewed anonymously in an efficient way. In terms of limitations, they are strictly related to the study design and the impossibility of verifying the correctness of some items (i.e., vaccinations undertaken). Nevertheless, we can conclude that there is a need for novel approaches in this field, and not only those based on education and training [35]. According to Corace and Garber, the behaviour change theories and principles could be used as a framework to plan, guide, and assess vaccine promotion interventions, with the aim of enhancing vaccine coverage among HCWs [36].

## 5. Conclusions

According to Maltezou and Poland [37], the immunization of HCWs represents a critical step toward patient safety. A vaccinated HCW protects him/herself and other people (patients, colleagues, relatives). In the case of oncology patients, people with multiple trauma or immunocompromised patients, having a vaccinated HCW could mean avoiding the worsening of their clinical conditions and increased public health expenditure.

## Figures and Tables

**Table 1:** Percentages of positive (agreement) answers on knowledge, attitude and behaviour.

Dependent variable	Measles	Mumps	Rubella	Varicella	Pertussis	Hepatitis B	Influenza	Tuberculosis
Which infectious diseases could be a risk for my health, if I am not vaccinated?	45.5	41.9	44.8	34	36.4	80	24	76
Is exposure to the disease a concrete risk factor for the patient’s health?	52.5	46.9	50.4	46.1	49.2	70.9	46.2	79.2
Does the disease X vaccine pose a risk to my health?	8.4	8.8	8.4	8.6	7.4	11.4	14.7	14.9
Are the benefits of the disease X vaccine higher than the risks for me?	61.1	56.9	60.4	54.3	57.8	73.2	45.7	60.4
Will the disease X vaccine protect my patients?	61.8	57.8	62	56.7	56.4	70.1	54.1	60.8
For which disease have I been vaccinated?	62.7	55.9	57.8	40.3	48.7	82	28.5	42.4
For which disease is the National health system providing sufficient communication/information?	17.7	13.5	14.9	11.7	11.2	21.9	37.1	14.2
For which diseases do you believe a vaccination should be mandatory for health care workers?	38.4	37	40.3	33.8	39.8	77.8	40.3	64.8
For which diseases do you believe a vaccination should be mandatory for biomedical students?	38.2	35	38.9	33.5	38.7	75.7	36.6	62.9
For which diseases do you believe a vaccination should be highly recommended, like for health care workers and students?	39.6	37.1	40.8	34.9	41.5	71.5	46.8	60.9

**Table 2:** Results of the multivariate analysis considering Hepatitis B.

Dependent Variable	Age	Years of Work	Nurses	Other Health Professions	Students	Females	Public Employment
Which infectious disease could be a risk for my health, if I am not vaccinated?	**0.95** **(0.90–0.99)**	**1.05** **(1–1.11)**	**0.18** **(0.06–0.51)**	**0.25** **(0.09–0.69)**	0.53 (0.09–3.20)	**1.93** **(1.12–3.33)**	1.11 (0.63–1.95)
Is exposure to the disease a concrete risk factor for the patient’s health?	**0.95** **(0.90–0.99)**	**1.06** **(1–1.11)**	**0.31** **(0.14–0.69)**	**0.35** **(0.17–0.72)**	0.79 (0.21–2.97)	1.49 (0.92–2.39)	0.79 (0.48–1.30)
Does the disease X vaccine pose a risk to my health?	**1.01** **(0.92–1.02)**	1.03 (0.98–1.08)	**0.54** **(0.25–1.18)**	**3.05** **(0.86–10.80)**	1.30 (0.11–14.36)	1.08 (0.50–2.32)	1.63 (0.71–3.71)
Are the benefits of the disease X vaccine higher than the risks for me?	**0.97** **(0.90–0.98)**	1.04 (0.99–1.09)	**0.18** **(0.08–0.42)**	**0.28** **(0.12–0.61)**	0.25 (0.07–3.69)	1.28 (0.78–2.10)	0.74 (0.44–1.25)
Will the disease X vaccine protect my patients?	**0.94** **(0.90–0.98)**	1.04 (0.99–1.09)	**0.18** **(0.08–0.41)**	**0.28** **(0.12–0.61)**	**0.25** **(0.07–0.90)**	1.15 (0.72–1.86)	1.10 (0.68–1.80)
For which disease have I been vaccinated?	0.98 (0.93–1.03)	1.01 (0.96–1.07)	0.70 (0.29–1.66)	0.55 (0.25–1.21)	0.36 (0.10–1.28)	**1.75** **(1.03–2.98)**	1.07 (0.61–1.89)
Did you show side effects after the vaccination for disease X?	0.99 (0.82–1.18)	1.02 (0.84–1.23)	1.04 (0.08–13.18)	0.86 (0.08–9.27)		1.22 (0.20–7.20)	0.53 (0.61–2.85)
For which diseases is the National health system providing sufficient communication/information?	0.98 (0.92–1.04)	1 (0.93–1.06)	0.56 (0.24–1.31)	0.49 (0.23–1.06)	1.54 (0.49–4.83)	1.16 (0.63–2.15)	**2.34** **(1.10–4.97)**
For which diseases do you believe a vaccination should be mandatory for health care workers?	**0.93** **(0.88–0.98)**	**1.07** **(1.01–1.13)**	**0.19** **(0.08–0.44)**	**0.28** **(0.12–0.62)**		1.62 (0.98–2.69)	**0.59** **(0.35–1)**
For which diseases do you believe a vaccination should be mandatory for biomedical students?	**0.94** **(0.90–1)**	**1.05** **(1–1.10)**	**0.19** **(0.08–0.44)**	**0.25** **(0.11–0.55)**	1.13 (0.21–6.04)	**1.87** **(1.14–3.04)**	0.72 (0.43–1.20)
For which diseases do you believe a vaccination should be highly recommended, like for health care workers and students?	**0.96** **(0.92–1)**	**1.05** **(1–1.11)**	**0.44** **(0.21–0.92)**	**0.39** **(0.20–0.77)**	0.86 (0.27–2.78)	**1.77** **(1.10–2.84)**	**0.42** **(0.25–0.70)**

The bold is referring to the OR that are statistically significant.

**Table 3:** Results of the multivariate analysis considering TB.

Dependent Variable	Age	Years of Work	Nurses	Other Health Professions	Students	Females	Public Employment
Which infectious diseases could be a risk for my health, if I am not vaccinated?	1 (0.94–1.04)	1 (0.95–1.06)	0.64 (0.30–1.39)	0.73 (0.36–1.48)	1.18 (0.31–4.44)	**2.13** **(1.29–3.52)**	0.85 (0.49–1.45)
Is exposure to the disease a concrete risk factor for the patient’s health?	**0.95** **(0.91–1)**	1 (0.99–1.10)	0.75 (0.32–1.75)	**0.47** **(0.22–1)**	0.51 (0.14–1.84)	1.26 (0.75–2.12)	0.72 (0.41–1.26)
Does the disease X vaccine pose a risk to my health?	0.98 (0.91–1.05)	1 (0.92–1.06)	0.86 (0.29–2.52)	1.45 (0.58–3.65)	0.52 (0.09–2.98)	1.41 (0.69–2.87)	1.74 (0.83–3.61)
Are the benefits of the disease X vaccine higher than the risks for me?	0.98 (0.94–1.02)	1.03 (0.98–1.08)	0.87 (0.44–1.70)	0.66 (0.36–1.21)	1.91 (0.59–6.18)	0.96 (0.61–1.50)	1.03 (0.65–1.63)
Will the disease X vaccine protect my patients?	1 (0.95–1.03)	1 (0.96–1.05)	0.84 (0.44–1.61)	1 (0.55–1.80)	1.63 (0.55–4.84)	1.25 (0.81–1.95)	1 (0.95–1.03)
For which disease have I been vaccinated?	1.01 (0.96–1.05)	1 (0.96–1.05)	1 (0.51–1.93)	0.96 (0.53–1.75)	2.31 (0.80–6.59)	1.20 (0.77–1.87)	0.94 (0.59–1.49)
Did you show side effects after the vaccination for disease X?	1.13 (0.83–1.52)	0.91 (0.67–1.24)				0.38 (0.02–6.74)	
For which diseases is the National health system providing sufficient communication/information?	1 (0.92–1.06)	1 (0.93–1.08)	0.51 (0.17–1.49)	0.64 (0.26–1.57)	2.06 (0.55–7.68)	0.98 (0.47–2.01)	1.58 (0.67–3.72)
For which diseases do you believe a vaccination should be mandatory for health care workers?	1 (0.95–1.04)	0.98 (0.93–1.03)	0.94 (0.49–1.82)	1.35 (0.74–2.46)	**6.31** **(1.29–30.85)**	**1.61** **(1.02–2.54)**	0.96 (0.60–1.54)
For which diseases do you believe a vaccination should be mandatory for biomedical students?	1 (0.96–1.05)	0.97 (0.92–1.02)	1.12 (0.57–2.20)	0.97 (0.53–1.77)	2.38 (0.67–8.41)	**1.58** **(1.01–2.47)**	1.08 (0.68–1.73)
For which diseases do you believe a vaccination should be highly recommended, like for health care workers and students?	1 (0.96–1.04)	1 (0.94–1.03)	1.50 (0.78–2.88)	1.36 (0.76–2.45)	2.25 (0.76–6.68)	1.22 (0.79–1.90)	0.88 (0.56–1.40)

The bold is referring to the OR that are statistically significant.

**Table 4:** Results of the multivariate analysis considering measles.

Dependent Variable	Age	Years of Work	Nurses	Other Health Professions	Students	Females	Public Employment
Which infectious diseases could be a risk for my health, if I am not vaccinated?	0.96 (0.92–1.01)	1.03 (0.98–1.08)	0.55 (0.30–1.01)	**0.48** **(0.24–0.93)**	0.66 (0.22–1.91)	1 (0.94–1.04)	**1.91** **(1.19–3.04)**
Is exposure to the disease a concrete risk factor for the patient’s health?	0.97 (0.93–1.01)	1.03 (0.98–1.08)	**0.34** **(0.17–0.67)**	**0.28** **(0.15–0.54)**	**0.27** **(0.09–0.74)**	1.22 (0.78–1.92)	1.25 (0.79–1.97)
Does the disease X vaccine pose a risk to my health?	**1.09** **(1.02–1.16)**	**0.90** **(0.84–0.97)**	1.49 (0.40–5.44)	2.21 (0.70–6.89)	1 (0.09–10.47)	0.84 (0.38–1.83)	1.26 (0.55–288)
Are the benefits of the disease X vaccine higher than the risks for me?	0.97 (0.93–1.01)	1.03 (0.98–1.08)	**0.36** **(0.19–0.68)**	**0.37** **(0.20–0.66)**	1.19 (0.33–4.30)	1.36 (0.86–2.14)	1.26 (0.80–2)
Will the disease X vaccine protect my patients?	**0.94** **(0.90–0.99)**	1.04 (0.99–1.09)	**0.16** **(0.07–0.37)**	**0.18** **(0.08–0.38)**	**0.24** **(0.07–0.80)**	1.05 (0.66–1.66)	1.38 (0.86–2.20)
For which disease have I been vaccinated?	**0.95** **(0.93–0.97)**	**1.04** **(1–1.09)**	**0.18** **(0.08–0.40)**	**0.19** **(0.09–0.41)**	**0.22** **(0.06–0.75)**	0.89 (0.56–1.42)	**1.75** **(1.08–2.83)**
Did you show side effects after the vaccination for the disease X?	1.12 (0.86–1.46)	0.89 (0.67–1.17)				0.80 (0.04–14.10)	
For which diseases is the National health system providing sufficient communication/information?	0.98 (0.92–1.05)	1.02 (0.95–1.09)	**0.39** **(0.16–0.94)**	0.50 (0.25–1.01)	1.18 (0.34–4.09)	1.27 (0.66–2.45)	2.06 (0.93–4.55)
For which diseases do you believe a vaccination should be mandatory for health care workers?	**0.93** **(0.88–0.98)**	**1.07** **(1.01–1.13)**	**0.13** **(0.06–0.27)**	**0.29** **(0.16–0.51)**	0.91 (0.28–2.96)	**1.07** **(1.01–1.13)**	**2.51** **(1.44–4.34)**
For which diseases do you believe a vaccination should be mandatory for biomedical students?	**0.92** **(0.87–0.97)**	**1.08** **(1.02–1.15)**	**0.17** **(0.09–0.34)**	**0.25** **(0.14–0.45)**	1.35 (0.39–4.57)	1.14 (0.69–1.87)	**2.06** **(1.21–3.51)**
For which diseases do you believe a vaccination should be highly recommended, like for health care workers and students?	**0.93** **(0.89–0.98)**	**1.06** **(1.01–1.12)**	**0.25** **(0.13–0.47)**	**0.36** **(0.21–0.63)**	0.61 (0.20–1.83)	1 (0.61–1.60)	1.33 (0.80–2.23)

The bold is referring to the OR that are statistically significant.

**Table 5:** Results of the multivariate analysis considering influenza.

Dependent Variable	Age	Years of Work	Nurses	Other Health Professions	Students	Females	Public Employment
Which infectious diseases could be a risk for my health, if I am not vaccinated?	1 (0.94–1.04)	1.02 (0.97–1.08)	0.53 (0.26–1.08)	**0.53** **(0.29–1)**	0.45 (0.13–1.49)	0.86 (0.53–1.41)	**1.86** **(1.08–3.22)**
Is exposure to the disease a concrete risk factor for the patient’s health?	0.98 (0.93–1.02)	1.02 (0.98–1.07)	0.64 (0.33–1.24)	**0.43** **(0.23–0.78)**	0.41 (0.14–1.18)	0.96 (0.62–1.50)	0.98 (0.93–1.02)
Does the disease X vaccine pose a risk to my health?	1.04 (0.98–1.10)	0.95 (0.89–1.01)	1.93 (0.69–5.36)	1.53 (0.58–4)		1.48 (0.76–2.88)	**0.53** **(0.28–0.97)**
Are the benefits of the disease X vaccine higher than the risks for me?	1.03 (0.98–1.07)	1 (0.94–1.04)	**0.45** **(0.22–0.89)**	**0.31** **(0.17–0.59)**	1.14 (0.39–3.33)	0.68 (0.43–1.07)	0.78 (0.48–1.25)
Will the disease X vaccine protect my patients?	1 (0.95–1.04)	1.01 (0.97–1.06)	0.58 (0.29–1.17)	**0.34** **(0.18–0.64)**	0.37 (0.12–1.07)	1.03 (0.66–1.61)	0.79 (0.49–1.26)
For which disease have I been vaccinated?	1.02 (0.98–1.07)	1 (0.95–1.05)	**0.36** **(0.18–0.68)**	**0.34** **(0.19–0.60)**	0.57 (0.19–1.69)	0.80 (0.50–1.30)	**2.15** **(1.25–3.68)**
Did you show side effects after the vaccination for the disease X?	0.94 (0.57–1.54)	0.81 (0.40–1.64)					
For which diseases is the National health system providing sufficient communication/information?	1 (0.94–1.03)	1 (0.96–1.05)	0.64 (0.33–1.24)	**0.55** **(0.30–1)**	0.94 (0.33–2.66)	0.70 (0.45–1.09)	1.32 (0.83–2.12)
For which diseases do you believe a vaccination should be mandatory for health care workers?	1 (0.94–1.03)	1.01 (0.96–1.06)	**0.12** **(0.05–0.25)**	**0.15** **(0.07–0.29)**	0.46 (0.15–1.38)	0.91 (0.57–1.47)	1.04 (0.63–1.71)
For which diseases do you believe a vaccination should be mandatory for biomedical students?	1 (0.95–1.04)	1 (0.96–1.06)	**0.19** **(0.09–0.38)**	**0.17** **(0.09–0.32)**	**0.32** **(0.11–0.96)**	1.43 (0.88–2.31)	1.01 (0.61–1.65)
For which diseases do you believe a vaccination should be highly recommended, like for health care workers and students?	1 (0.95–1.04)	1 (0.96–1.05)	**0.36** **(0.18–0.70)**	**0.38** **(0.21–0.71)**	0.92 (0.31–2.71)	0.96 (0.62–1.50)	1.08 (0.68–1.71)

The bold is referring to the OR that are statistically significant.

**Table 6:** Results of the multivariate analysis considering varicella.

Dependent Variable	Age	Years of Work	Nurses	Other Health Professions	Students	Females	Public Employment
Which infectious diseases could be a risk for my health, if I am not vaccinated?	0.99 (0.97–1.01)	0.97 (0.93–1.02)	0.66 (0.35–1.25)	0.61 (0.34–1.08)	**0.44** **(0.23–0.85)**	1.16 (0.79–1.69)	**2.18** **(1.34–3.54)**
Is exposure to the disease a concrete risk factor for the patient’s health?	0.99 (0.97–1.01)	1.03 (0.98–1.08)	0.59 (0.32–1.11)	**0.50** **(0.28–0.89)**	**0.45** **(0.23–0.84)**	1.12 (0.78–1.60)	1.43 (0.92–2.23)
Does the disease X vaccine pose a risk to my health?	0.98 (0.95–1.02)	**0.89** **(0.82–0.96)**	1.76 (0.43–7.16)	2.74 (0.77–9.72)	1.93 (0.50–7.41)	1.24 (0.65–2.39)	2.11 (0.86–5.14)
Are the benefits of the disease X vaccine higher than the risks for me?	0.99 (0.95–1.03)	1.01 (0.96–1.06)	0.55 (0.28–1.07)	**0.46** **(0.25–0.85)**	1.71 (0.52–5.58)	1.36 (0.87–2.12)	1.20 (0.76–1.89)
Will the disease X vaccine protect my patients?	**0.94** **(0.90–0.99)**	**1.04** **(1–1.10)**	**0.26** **(0.13–0.54)**	**0.29** **(0.15–0.56)**	0.41 (0.13–1.29)	1.18 (0.75–1.86)	1.47 (0.93–2.34)
For which disease have I been vaccinated?	1 (0.95–1.04)	0.96 (0.91–1.01)	**2.90** **(1.32–6.36)**	**3.55** **(1.72–7.32)**	**4.63** **(1.50–14.24)**	0.66 (0.41–1.06)	2.13 (1.28–3.54)
Did you show side effects after the vaccination for the disease X?	1.12 (0.86–1.46)	0.89 (0.67–1.17)	1.05 (0.06–17.59)			0.80 (0.04–14.10)	
For which diseases is the National health system providing sufficient communication/information?	0.96 (0.88–1.06)	1.01 (0.92–1.12)	0.16 (0.03–0.80)	0.52 (0.19–1.36)	1.43 (0.35–5.74)	0.71 (0.31–1.65)	10.73 (1.39–82.32)
For which diseases do you believe a vaccination should be mandatory for health care workers?	0.94 (0.89–0.99)	1.06 (1–1.12)	0.12 (0.05–0.27)	0.31 (0.17–0.59)	1.31 (0.41–4.20)	0.93 (0.56–1.57)	2.48 (1.38–4.47)
For which diseases do you believe a vaccination should be mandatory for biomedical students?	0.93 (0.88–0.99)	1.07 (1.01–1.13)	0.18 (0.08–0.37)	0.28 (0.15–0.52)	1.11 (0.35–3.45)	1.10 (0.66–1.83)	1.95 (1.11–3.43)
For which diseases do you believe a vaccination should be highly recommended, like for health care workers and students?	0.92 (0.87–0.98)	1.07 (1.01–1.14)	0.32 (0.15–0.68)	0.42 (0.22–0.80)	0.82 (0.27–2.49)	0.83 (0.51–1.37)	1.67 (0.96–2.90)

The bold is referring to the OR that are statistically significant.

**Table 7:** Results of the multivariate analysis considering pertussis.

Dependent Variable	Age	Years of Work	Nurses	Other Health Professions	Students	Females	Public Employment
Which infectious diseases could be a risk for my health, if I am not vaccinated?	0.97 (0.92–1.01)	1 (0.95–1.05)	**0.48** **(0.24–0.94)**	**0.50** **(0.27–0.92)**	0.69 (0.24–2.01)	0.73 (0.46–1.16)	1.38 (0.85–2.24)
Is exposure to the disease a concrete risk factor for the patient’s health?	**0.96** **(0.92–1)**	1.03 (0.98–1.08)	**0.42** **(0.21–0.82)**	**0.29** **(0.15–0.54)**	0.43 (0.14–1.26)	1.06 (0.68–1.65)	0.78 (0.49–1.25)
Does the disease X vaccine pose a risk to my health?	**1.07** **(1.01–1.15)**	**0.90** **(0.83–0.97)**	2.49 (0.62–10.03)	2.35 (0.64–8.64)	1 (0.09–10.95)	0.96 (0.41–2.26)	2.31 (0.87–6.09)
Are the benefits of the disease X vaccine higher than the risks for me?	0.98 (0.94–1.02)	1.02 (0.97–1.06)	0.73 (0.38–1.43)	**0.51** **(0.28–0.94)**	1.37 (0.44–4.22)	1.09 (0.70–1.70)	1.07 (0.68–1.68)
Will the disease X vaccine protect my patients?	0.94 (0.90–0.98)	1.04 (0.99–1.09)	**0.21** **(0.10–0.46)**	**0.20** **(0.10–0.42)**	**0.21** **0.06–0.70)**	0.87 (0.55–1.38)	1.12 (0.70–1.79)
For which disease have I been vaccinated?	0.98 (0.93–1.02)	0.97 (0.92–1.02)	1.56 (0.79–3.09)	1.34 (0.72–2.48)	1.21 (0.41–3.50)	0.87 (0.55–1.37)	**1.91** **(1.18–3.09)**
Did you show side effects after the vaccination for the disease X?	1.12 (0.86–1.46)	0.89 (0.67–1.17)	1.05 (0.06–17.59)			0.80 (0.04–14.10)	
For which diseases is the National health system providing sufficient communication/information?	1 (0.93–1.08)	0.99 (0.91–1.07)	**0.15** **(0.03–0.75)**	0.67 (0.27–1.67)	2.27 (0.59–8.65)	0.60 (0.27–1.31)	2.03 (0.72–5.73)
For which diseases do you believe a vaccination should be mandatory for health care workers?	**0.92** **(0.87–0.98)**	**1.07** **(1.01–1.13)**	**0.14** **(0.06–0.31)**	**0.34** **(0.18–0.63)**	1.52 (0.45–5.20)	0.78 (0.47–1.30)	**2.11** **(1.21–3.69)**
For which diseases do you believe a vaccination should be mandatory for biomedical students?	**0.93** **(0.88–0.98)**	**1.07** **(1.01–1.13)**	**0.21** **(0.10–0.42)**	**0.28** **(0.15–0.52)**	1.24 (0.38–3.98)	0.94 (0.58–1.52)	1.32 (0.79–2.21)
For which diseases do you believe a vaccination should be highly recommended, like for health care workers and students?	**0.92** **(0.88–0.97)**	**1.08** **(1.02–1.14)**	**0.50** **(0.25–0.98)**	0.64 (0.35–1.17)	1.14 (0.38–3.39)	0.78 (0.50–1.24)	1.03 (0.64–1.66)

The bold is referring to the OR that are statistically significant.

**Table 8:** Results of the multivariate analysis considering mumps.

Dependent Variable	Age	Years of Work	Nurses	Other health Professions	Students	Females	Public Employment
Which infectious diseases could be a risk for my health, if I am not vaccinated?	0.98 (0.94–1.03)	1.01 (0.96–1.06)	0.70 (0.37–1.35)	0.61 (0.34–1.11)	1.54 (0.53–4.45)	0.79 (0.51–1.24)	1.13 (0.71–1.79)
Is exposure to the disease a concrete risk factor for the patient’s health?	**0.96** **(0.92–1)**	1.03 (0.98–1.08)	0.60 (0.31–1.16)	**0.38** **(0.21–0.70)**	0.61 (0.21–1.75)	1.13 (0.72–1.76)	1.01 (0.64–1.60)
Does the disease X vaccine pose a risk to my health?	**1.07** **(1–1.14)**	**0.93** **(0.86–1)**	1.54 (0.42–5.63)	1.93 (0.61–6.11)	0.88 (0.08–9.03)	1.26 (0.54–2.90)	2.38 (0.91–6.20)
Are the benefits of the disease X vaccine higher than the risks for me?	0.98 (0.94–1.02)	1.02 (0.98–1.07)	**0.43** **(0.22–0.86)**	**0.42** **(0.22–0.79)**	1.90 (0.53–6.7)	1 (0.64–1.56)	1.05 (0.67–1.67)
Will the disease X vaccine protect my patients?	**0.94** **(0.90–0.99)**	1.04 (0.99–1.10)	**0.20** **(0.09–0.43)**	**0.22** **(0.10–0.45)**	**0.24** **(0.07–0.76)**	1 (0.63–1.58)	1.38 (0.87–2.20)
For which disease have I been vaccinated?	**0.96** **(0.92–1)**	1 (0.95–1.05)	1.53 (0.79–3)	1.31 (0.71–2.4)	3.25 (0.93–11.36)	0.90 (0.95–1.05)	**1.61** **(1.01–2.58)**
Did you show side effects after the vaccination for the disease X?	**1.20** **(1.02–1.42)**	**0.81** **(0.65–1)**	0.93 (0.05–16.25)	0.35 (0.01–6.30)		1.10 (0.08–14.16)	1.06 (0.08–13.45)
For which disease is the National health system providing sufficient communication/information?	0.94 (0.86–1.02)	1.05 (0.96–1.16)	**0.21** **(0.06–0.72)**	0.53 (0.22–1.25)	1.13 (0.29–4.36)		2.16 (0.86–1.02)
For which diseases do you believe a vaccination should be mandatory for health care workers?	**0.94** **(0.89–0.99)**	**1.06** **(1–1.12)**	**0.20** **(0.09–0.41)**	**0.32** **(0.17–0.60)**	1.49 (0.47–4.73)	0.87 (0.52–1.44)	**2.75** **(1.52–4.96)**
For which diseases do you believe a vaccination should be mandatory for biomedical students?	**0.93** **(0.88–0.98)**	**1.07** **(1.01–1.14)**	**0.23** **(0.11–0.48)**	**0.33** **(0.18–0.62)**	1.64 (0.51–5.26)	1.02 (0.62–1.69)	2.02 (0.88–0.98)
For which diseases do you believe a vaccination should be highly recommended, like for health care workers and students?	**0.94** **(0.89–0.99)**	1.05 (0.99–1.11)	**0.31** **(0.15–0.63)**	**0.44** **(0.24–0.82)**	0.80 (0.27–2.37)	0.96 (0.59–1.57)	1.54 (0.91–2.61)

The bold is referring to the OR that are statistically significant.

**Table 9:** Results of the multivariate analysis considering rubella.

Dependent Variable	Age	Years of Work	Nurses	Other Health Professions	Students	Females	Public Employment
Which infectious diseases could be a risk for my health, if not vaccinated?	**0.96** **(0.92–1)**	1.01 (0.96–1.06)	0.61 (0.31–1.19)	0.77 (0.42–1.40)	0.97 (0.33–2.86)	1.32 (0.84–2.07)	1.59 (0.99–2.54)
Is exposure to the disease a concrete risk factor for the patient’s health?	0.97 (0.93–1.01)	1.02 (0.97–1.07)	0.61 (0.31–1.17)	0.58 (0.32–1.05)	0.53 (0.18–1.51)	1.28 (0.83–1.99)	0.95 (0.60–1.50)
Does the disease X vaccine pose a risk to my health?	1.07 (0.99–1.14)	0.93 (0.86–1.01)	0.72 (0.13–3.78)	2.94 (0.82–10.49)		1.95 (0.77–4.96)	1.17 (0.50–2.75)
Are the benefits of the disease X vaccine higher than the risks for me?	0.97 (0.93–1.02)	1.03 (0.98–1.08)	**0.38** **(0.18–0.77)**	**0.40** **(0.21–0.77)**	1.30 (0.36–4.66)	1.19 (0.76–1.87)	1.13 (0.71–1.80)
Will the disease X vaccine protect my patients?	**0.95** **(0.91–0.99)**	1.04 (0.99–1.09)	**0.22** **(0.10–0.47)**	**0.28** **(0.14–0.58)**	**0.27** **(0.08–0.88)**	1.17 (0.74–1.85)	0.95 (0.09–1.99)
For which disease have I been vaccinated?	**0.94** **(0.90–0.99)**	1 (0.95–1.05)	1.56 (0.79–3.10)	1.68 (0.90–3.13)	1.61 (0.51–5.07)	1.26 (0.80–1.99)	**1.87** **(1.15–3.02)**
Did you show side effects after the vaccination for the disease X?	1.12 (0.86–1.46)	0.89 (0.67–1.17)	1.05 (0.06–17.59)			0.80 (0.04–14.10)	
For which disease is the National health system providing sufficient communication/information?	0.95 (0.88–1.03)	1.04 (0.96–1.13)	**0.27** **(0.08–0.86)**	0.68 (0.29–1.56)	1.20 (0.32–4.42)	1.09 (0.54–2.19)	2.35 (0.97–5.67)
For which diseases do you believe a vaccination should be mandatory for health care workers?	**0.94** **(0.90–0–99)**	**1.05** **(1–1.11)**	**0.11** **(0.05–0.25)**	**0.31** **(0.17–0.58)**	1.06 (0.33–3.36)	0.97 (0.59–1.60)	**1.96** **(1.15–3.35)**
For which diseases do you believe a vaccination should be mandatory for biomedical students?	**0.95** **(0.90–1)**	1.05 (0.99–1.10)	**0.17** **(0.08–0.36)**	**0.31** **(0.17–0.57)**	1.62 (0.49–5.34)	0.98 (0.60–1.59)	**1.73** **(1.03–2.92)**
For which diseases do you believe a vaccination should be highly recommended, like for health care workers and students?	**0.93** **(0.88–0.98)**	**1.06** **(1–1.12)**	**0.32** **(0.15–0.64)**	**0.50** **(0.27–0.92)**	0.79 (0.26–2.33)	1.16 (0.72–1.86)	1.25 (0.88–0.98)

The bold is referring to the OR that are statistically significant.
